# Immunopathogenesis of Sarcoidosis

**DOI:** 10.1055/a-2716-5737

**Published:** 2025-11-06

**Authors:** Christen Vagts, Christian Ascoli, Jeffrey R. Jacobson

**Affiliations:** 1 Division of Pulmonary Critical Care Sleep and Allergy, Department of Medicine, University of Illinois Chicago, Chicago, Illinois, United States

**Keywords:** sarcoidosis, immunopathogenesis, granulomatous inflammation, innate immunity, adaptive immunity, interstitial lung disease

## Abstract

Sarcoidosis is a granulomatous disease of unknown cause, triggered by an unidentified antigen. Although classically considered a T cell–mediated disorder with an IFN-γ signature driven by Th1, Th17, and Th17.1 cells, its pathogenesis reflects dysregulated crosstalk between innate and adaptive immunity. Granulomas form through macrophage differentiation at the core, fueled by aberrantly programmed monocytes and sustained by persistent antigen presentation to T cells. Hyperactive macrophages drive excessive peripheral cell recruitment, while dysregulated T cell responses promote T cell expansion, impaired effector regulation, and eventual exhaustion. Deficient regulatory pathways fail to counterbalance this activation, creating a perpetuating inflammatory loop that underlies disease persistence and fibrotic progression. This review integrates up-to-date transcriptomic and biological data to define the cellular and molecular mechanisms that initiate, sustain, and dysregulate immune responses in sarcoidosis.

## Introduction

Sarcoidosis is a heterogeneous, multisystem, immune-mediated disease of unknown etiology. Although it occurs globally, with notable regional and ethnic variation, it is more prevalent among individuals of Scandinavian or African American descent and is observed more frequently in women. 1 2 The age of onset follows a bimodal distribution, peaking between 30 to 40 and 50 to 60 years, with older patients more likely to be women. 2 Intrathoracic involvement, affecting the lung and/or lymph nodes, occurs in up to 90% of cases, but extrapulmonary manifestations are also common with the skin, eyes, and/or heart, among others, affected. 3 Clinical manifestations are therefore highly variable and reflect the pattern and extent of organs involved.

Acute presentations such as Löfgren's syndrome, which is characterized by fever, bilateral hilar lymphadenopathy, and either ankle arthritis or erythema nodosum, are associated with an excellent prognosis, with most cases spontaneously resolving within 24 months. 1 In contrast, approximately 10 to 40% of patients develop chronic or progressive disease, which can result in irreversible organ dysfunction and fibrosis. 2 4 5 These demographic and clinical features underscore the complexity of sarcoidosis pathogenesis and highlight the significant heterogeneity in presentation and outcome, which has long been a barrier to standardized research classification.

The histopathologic hallmark of sarcoidosis is the presence of discrete, well-formed, non-necrotizing granulomas composed of epithelioid histiocytes and multinucleated giant cells (MGCs) surrounded by lymphocytes, plasma cells, and fibroblasts, which form in response to an unidentified antigen. 1 Although the precise trigger remains unknown, two complementary hypotheses of disease pathogenesis have emerged. One suggests a normal immune response to a persistent, uncleared, or poorly degraded microbial antigen or auto-antigen. The other proposes a pathogenic immune response marked by sustained activation, dysfunctional regulation, and impaired resolution. 1

**Fig. 1:**
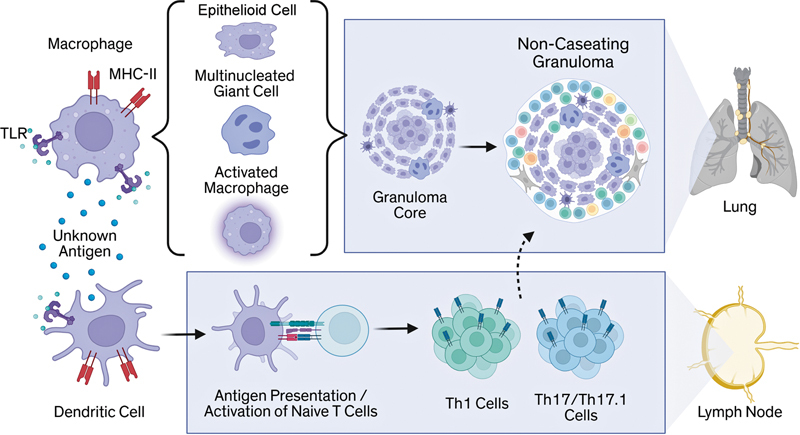
Model of granuloma formation. Following recognition of an unknown antigen via pattern recognition receptors such as toll-like receptors (TLRs), macrophages become activated, differentiate into epithelioid cells, and fuse to form multinucleated giant cells, establishing the granuloma core. In parallel, dendritic cells recognize and present antigen via MHC-II to naïve T cells in the lymph node, promoting their activation and differentiation into Th1 and Th17/Th17.1 effector subsets. These activated T cells migrate to the lung, where they release cytokines and chemokines that amplify macrophage activation and drive granuloma assembly. Created in BioRender. Vagts, C. (2025) https://BioRender.com/xjfj4le .

**Fig. 2:**
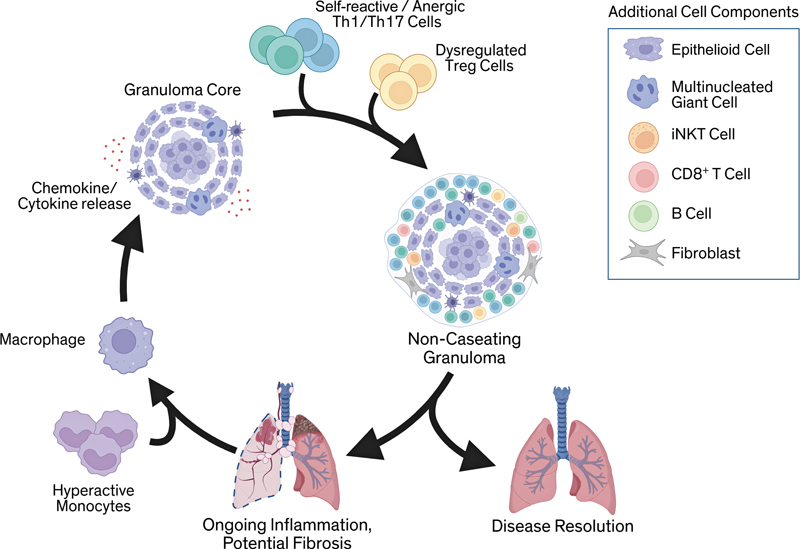
Model of granuloma maintenance. Hyperactivated and inflammatory macrophages form the granuloma core and secrete proinflammatory mediators, including IFN-γ, TNF-α, IL-1β, and additional chemokines that recruit activated T cells to local tissue, fueling granulomatous inflammation. This process is compounded by impaired regulatory control from T regulatory (Treg) cells. Hyperactive, dysregulated monocytes are also recruited to sites of inflammation, where they differentiate into alveolar macrophages, further driving inflammation in a perpetuating cycle. Additional components, including invariant natural killer T (iNKT) cells, CD8⁺ T cells, B cells, and fibroblasts, contribute to the granuloma structure and microenvironment. Patients may have persistent inflammation and subsequent fibrosis or spontaneous resolution. Created in BioRender. Vagts, C. (2025) https://BioRender.com/39h3z9v .

In this review, we explore both hypotheses and synthesize the current understandings of the complex and multifactorial immunologic mechanisms that underly sarcoidosis. Although traditionally considered a CD4 ^+^ T cell–mediated disease with a T-helper (Th)1, Th17, and Th17.1 signature, emerging evidence from mechanistic studies and novel -omics approaches have broadened this perspective to include widespread dysregulation of both innate and adaptive immunity. Abnormalities in monocyte and macrophage function, altered antigen presentation, and imbalances in effector and regulatory T cell subsets have all been described. Additional contributions from Th17 cells, B cells, and unconventional T cell populations further underscore the immunologic complexity of the disease. Figs. 1 and 2 outline proposed models of granuloma formation and maintenance, highlighting the cellular interactions that drive sarcoidosis pathogenesis. This review aims to integrate these diverse insights into a cohesive framework, highlight key immunologic pathways that are dysregulated in sarcoidosis, and underscore their potential to inform future therapeutic discovery.

## Disease Trigger

### Genetic Predisposition

Sarcoidosis susceptibility reflects an interplay between genetic predisposition and environmental or microbial exposures. Familial aggregation studies demonstrate a 3.7-fold increased risk among first-degree relatives with heritability estimated at 39%. 6 This supports a substantial genetic contribution, though familial prevalence varies widely across geographic regions. 7

Genome-wide association studies (GWAS) in sarcoidosis have identified multiple susceptibility loci, particularly within the HLA class II region including HLA-DRB1, DQA1, and DQB1, implicating antigen presentation to CD4 ^+^ T cells in pathogenesis. Associations are phenotype- and region-specific. For example, HLA-DRB1*0301 correlates with Löfgren's syndrome; HLA-DRB*12 and *14 with pulmonary sarcoidosis in the Netherlands 8 ; and HLA-DRB1*04 with extrapulmonary disease, including in combination with HLA-DRB*15 in Sweden 9 and with HLA- DQB1* 0301 in both Japanese and UK cohorts, 8 where strong links to uveitis and sarcoidosis-associated hypercalcemia have been observed. 6 8

Although informative, ancestry-restricted studies have limited generalizability and may fail to capture risk variants present in other populations, potentially overlooking both shared mechanisms and ancestry-specific contributors to disease. To address this gap, one of the largest multiethnic GWAS to date, conducted by Liao et al, examined genetic risk for sarcoidosis in European American and African American cohorts, and identified HLA alleles DRB1 * 0101, DQA1 * 0101, and DQB1*0501 as shared risk variants across both groups. 10 These findings reinforce the central role of HLA variation in modulating susceptibility, phenotype, and prognosis, while clarifying both common and ancestry-specific genetic determinants of sarcoidosis.

Several non-HLA susceptibility loci have been described, including IL23R, ATXN2/SH2B3, ANXA11, IL12B, and MANBA/NFKB1. 11 12 Functional studies and transcriptome-wide association analyses suggest that these genes participate in key immunologic processes relevant to sarcoidosis. Specifically, they are associated with dysregulation of the IL-23/Th17 axis, heightened T cell activation, and abnormal leukocyte adhesion and trafficking, mechanisms that have emerged as central features of sarcoidosis pathogenesis. 10 11 12

### Etiologic Antigens

Granuloma formation in sarcoidosis is initiated by an unidentified antigen, representing the immune system's attempt to contain a stimulus it cannot fully eliminate. Similar responses occur in infectious diseases with histologic overlap, such as tuberculosis. 13 Granulomas in sarcoidosis, however, are predominantly non-caseating and lack central necrosis, in contrast to the caseating granulomas of tuberculosis, where necrosis reflects the cytotoxicity of actively replicating *Mycobacterium tuberculosis* and subsequent macrophage apoptosis. 13 Although antimycobacterial therapy has been associated with reduced granuloma burden in cutaneous sarcoidosis 14 and improved pulmonary function in pulmonary sarcoidosis, 15 the absence of necrosis in sarcoidosis suggests that the inciting antigen is non-viable, non-replicating, or of low immunogenicity.

Although no single antigen has been definitively established as a universal trigger, numerous microbial and self-derived candidates have been proposed. 16 These include pathogen-associated molecular patterns (PAMPs) derived from microbes and self-derived damage-associated molecular patterns (DAMPs) released from stressed or injured host cells, both of which may activate downstream immune responses.

A wide range of microbial components have been investigated as potential contributors to sarcoidosis pathogenesis. These include mycobacterial cell wall components, DNA, cytosolic proteins such as early secreted antigenic target 6 (ESAT-6) and culture filtrate protein 10 (CFP10), superoxide dismutase A (SODA), and various microbial heat shock proteins 14 which serve as PAMPs and trigger downstream inflammatory responses. Peripheral blood mononuclear cells (PBMCs) and bronchoalveolar lavage (BAL) cells from patients with sarcoidosis have demonstrated altered adaptive immune responses following exposure to these antigens, including ESAT-6 and mKatG. 17 18 19 Moreover, molecular analyses have identified both *Mycobacterium tuberculosis* and non-tuberculous mycobacterial DNA in sarcoidosis tissue samples, 1 supporting a potential role for persistent mycobacterial antigens in driving granulomatous inflammation.

Non-infectious mechanisms proposed in sarcoidosis pathogenesis include autoimmune responses to self-antigens or DAMPs, such as serum amyloid A (SAA), vimentin, and various environmental exposures. SAA is an acute phase apolipoprotein that modulates inflammation via NF-κB signaling. 20 It is highly expressed in sarcoidosis granulomas, 21 elevated in the serum of sarcoidosis patients, 22 23 correlates with treatment need, and may serve as a biomarker for disease. Vimentin, another prominent self-antigen implicated in sarcoidosis, is an intermediate filament protein upregulated in response to cellular stress, injury, and inflammation. Elevated anti-vimentin antibodies (AVA) in serum and bronchoalveolar lavage fluid (BALF), along with their association with vimentin-rich tertiary lymphoid structures in the lung, support a role for vimentin in ongoing immune activation and local granulomatous inflammation. 24 25 Further, Bagavant et al demonstrated that intratracheal instillation of vimentin-coated beads in mice induced granuloma formation and a sarcoidosis-like immune response, 26 implicating vimentin as a self-antigen capable of initiating granulomatous inflammation in the absence of infection. Finally, autoantibody reactivity to four proteins in BALF and serum, which varied by disease phenotype, suggests a broader autoantigenic landscape in sarcoidosis. 27

Environmental exposures such as metals, silica, and inorganic dust are associated with increased sarcoidosis risk, 28 though specific antigenic drivers remain incompletely defined. These agents may act as persistent inorganic antigens or adjuvants that amplify immune activation, promote granuloma formation, and, in some cases, drive fibrotic progression.

In addition to the above mechanisms, the respiratory microbiome likely influences sarcoidosis pathogenesis, reflecting a complex interplay between host immunity and microbial composition. Airway microbiome studies have shown shifts in microbial composition, including increased abundance of *Atopobium* , *Fusobacterium* , and various bacterial and fungal taxa in BALF. 29 30 31 32 *Propionibacterium acnes* has been localized within granulomas in over 88% of sarcoidosis lymph node samples using a species-specific monoclonal antibody, suggesting a possible etiologic role. 33 Additionally, increasing attention has been directed toward the gut–lung axis; a growing evidence suggests the gut microbiota can modulate systemic and pulmonary immune responses. 34 In sarcoidosis, alterations in microbial populations are observed in both the gastrointestinal and respiratory tracts, with loss of gut microbial diversity. 35 Collectively, these findings support a model in which dysbiosis in the lungs, gut, or both may act as a persistent immunologic trigger, driving granuloma formation and sustaining chronic inflammation in genetically susceptible individuals.

## Innate Immune Dysregulation

### Activation of Innate Immunity

PAMPs and DAMPs activate pattern recognition receptors (PRRs) such as Toll-like receptors (TLRs) and NOD-like receptors (NLRs) on innate immune cells, including macrophages, dendritic cells (DCs), and monocytes, to initiate innate immune responses that are critical to granuloma formation. 36 In sarcoidosis, PRR signaling is dysregulated across compartments. In the blood, TLR2, TLR4, and NOD2 are upregulated on circulating monocytes, and co-stimulation synergistically amplifies cytokine release, resulting in up to a 4-fold increase in TNF-α and a 13-fold increase in IL-1β. 37 38 In tissue, TLR2 and TLR4 expression is increased in cutaneous sarcoidosis lesions, 39 and TLR2 is also elevated in mediastinal lymph nodes. 40 In contrast, alveolar macrophages from BALF show decreased surface TLR2 expression compared with healthy controls, 41 yet paradoxically display excessive TNF-α and IL-6 production in response to TLR2/1 stimulation, and a blunted response to TLR2/6 ligation despite preserved TLR2 levels, 42 reflecting functional dysregulation. Additionally, in a *Propionibacterium acnes* mouse model, TLR2 deletion significantly attenuated granuloma formation, implicating TLR2 as a central driver of granulomatous inflammation. TLR2 signaling acts through NF-κB stimulated by SAA, 21 whereas evidence suggests that TLR4-mediated inflammation in sarcoidosis involves dysregulated p38 MAPK signaling, 43 leading to a heightened proinflammatory cytokine response. Together, these findings suggest that upregulation and dysregulation of TLR and NOD2 signaling contribute to persistent innate immune activation and granuloma maintenance in sarcoidosis.

### Macrophages: Granuloma Initiation and Effector Function

Macrophages are versatile innate immune cells that maintain tissue homeostasis through phagocytic function, while also serving as antigen-presenting cells to link innate and adaptive immunity. 44 Classically, activated M1 macrophages arise in response to Th1-derived cytokines such as IFN-γ and are characterized by a proinflammatory phenotype, promoting antigen presentation and cytokine production (e.g., TNF-α, IL-12). In contrast, alternatively activated M2 macrophages, induced by Th2-associated cytokines like IL-4 and IL-13, exhibit anti-inflammatory and tissue remodeling functions with the potential to promote fibrosis.

Macrophages are central to sarcoidosis immunopathogenesis. Following PRR stimulation, monocyte-derived macrophages are recruited to sites of inflammation where they differentiate into epithelioid histiocytes and MGCs, hallmark components of the granuloma core. Epithelioid cells form adherens junctions and interdigitated membranes, creating a physical barrier around the antigen, while MGCs retain phagocytic capabilities for ongoing antigen processing. 45 46 47 Beyond structural roles, macrophages drive disease progression through persistent cytokine secretion, antigen presentation, and immune recruitment.

Functionally, granuloma-associated macrophages display both proinflammatory and profibrotic programs. They exhibit increased spontaneous production of TNF-α and IL-1β, 48 49 which correlates with disease severity. 48 49 Heightened SAA-driven NLRP3 inflammasome activation has been identified as a key mechanism of IL-1β production, with granuloma formation attenuated in NLRP3-deficient mice. 50 Despite evidence of proinflammatory activation states, sarcoidosis has been characterized by a heterogeneous landscape, with evidence of both M1- and M2-like activation across disease stages and anatomical compartments. 41 51 52 53 54 Shamaei et al identified increased expression of CD163 and CD206 in lung and lymph node granulomas, consistent with alternatively activated macrophages with an M2 phenotype. ^52^ In an in vitro granuloma model, Locke et al demonstrated that IL-13–driven STAT6 signaling promoted CD163 ^+^ macrophage differentiation, suggesting that a Th2-skewed cytokine environment may contribute to alternative macrophage activation within sarcoid granulomas. 51 This profibrotic skew is further supported by increased expression of CCL18 in sarcoidosis BALF alveolar macrophages, a chemokine associated with fibrosis and poor prognosis in idiopathic pulmonary fibrosis. 55 56 Despite these M2-like and fibrotic features, Crouser et al showed that granulomas generated from sarcoidosis patient–derived PBMCs released higher levels of IFN-γ, TNF-α, IL-1β, and IL-10 compared with those from individuals with latent tuberculosis infection, underscoring their persistent proinflammatory potential. 57 These findings collectively support a context-dependent, transitional activation state in sarcoidosis macrophages. The coexistence of proinflammatory and profibrotic features highlights the plasticity of macrophage activation and underscores the limitations of applying a binary M1/M2 framework to granulomatous inflammation in sarcoidosis.

Mechanistically, impaired antigen clearance and sustained mTORC1 signaling underlie persistent macrophage activation in sarcoidosis. In a myeloid-specific knockout mouse model, Linke et al demonstrated that constitutive mTORC1 activation promotes granuloma initiation and persistence through increased macrophage proliferation, inhibition of apoptosis, and metabolic reprogramming, levels of which correlated with disease progression in sarcoidosis lung samples. 58 mTORC1 also promotes alternative M2 macrophage activation, as observed in human sarcoidosis tissues and in a complementary in vitro granuloma model. In this model, sarcoid granulomas exhibited enhanced phagolysosomal activity, persistent intracellular antigen processing, and upregulation of mTORC1/S6/STAT3 signaling compared with granulomas from latent tuberculosis infection. 57 Transcriptomic profiling revealed upregulation of genes involved in phagosome–lysosome fusion, antigen presentation, and innate immune sensing. Differentially expressed genes included those supporting microbial opsonization (C1QA, C1QB), pattern recognition (MRC1 [CD206], TREM1/TYROBP complex), scavenger receptor signaling (CD163), intracellular killing, and MHC class II–mediated T cell activation. Prior studies similarly demonstrated enhanced antigen presentation and T cell co-stimulation by alveolar macrophages in sarcoidosis. 59 60 Together, these findings highlight the essential role of mTORC1-driven metabolic and functional programming in granuloma formation and persistence in sarcoidosis.

Recent advances in spatial transcriptomics, single-cell RNA sequencing (scRNA-seq), and multiplex imaging have illuminated the central role of macrophages in orchestrating sarcoid granuloma architecture and immune function. In a multi-omic spatial and scRNA-seq analysis of cutaneous sarcoidosis granulomas, Krausgruber et al identified granuloma-associated (GA) macrophages and homeostatic macrophages as predominant macrophage subpopulations. GA macrophages exhibited a strong IFN-γ–driven activation profile, marked by high *IFNGR1* and IFN-γ–inducible chemokines, alongside enrichment in antigen presentation, lysosomal function, extracellular matrix (ECM) remodeling, and mTORC1 pathways. Canonical inflammatory mediators ( *IL1, IL10, TNF, NFKB* ) and apoptosis-related genes were downregulated, suggesting a non-classical, sustained activation state. Subclusters were characterized by differential expression of several classical sarcoidosis-related genes, including *ACE* and *CHIT1* , which serve as clinically recognized biomarkers of sarcoidosis, alongside marked upregulation of *MMP9* , *MMP12* , and *MMP14* , implicating these cells in tissue remodeling and potential fibrotic progression. Cross-cell communication mapping revealed spatially relevant ligand–receptor interactions, notably CXCL9-CXCR3 and CCL5-CCL1/CCR5 between macrophages and T cells, supporting macrophage-directed chemotactic recruitment and cellular organization within the granuloma microenvironment. 61 These findings suggest that granuloma-associated macrophages in sarcoidosis function as persistently activated proinflammatory orchestrators of immune cell recruitment and matrix remodeling that contribute to granuloma formation and maintenance.

Additional technologic advances including in situ sequencing and multiplex immunofluorescence have enabled high-resolution spatial analysis of granuloma structure and gene expression in sarcoidosis. Using these techniques, Carow et al showed that sarcoidosis granulomas lack the tertiary lymphoid structures and organized antigen-specific niches characteristic of tuberculosis, and are instead composed of myeloid-rich, lymphocyte-poor cores enriched in macrophage-associated transcripts including CD11b ( *ITGAM* ), CD11c ( *ITGAX* ), *CD14* , and *HLA-DRA* . 62 These findings suggest that sarcoidosis granulomas are maintained through distinct immunologic mechanisms, driven largely by macrophages and their microenvironment.

In addition, macrophages secrete chemokines such as macrophage inflammatory proteins MIP-1α, MIP-1β, and MIP-3β, 63 64 promoting immune cell recruitment and reinforcing their central role in sustaining the granulomatous response. These findings highlight macrophages as dynamic, plastic cells whose dysregulated behavior sustains chronic inflammation and contributes to sarcoidosis heterogeneity.

Together, these data assert macrophages as metabolically reprogrammed, persistently activated orchestrators of granuloma architecture and immune recruitment. Their sustained activation depends on continuous crosstalk with T cells, as this review will further highlight, which provides critical cytokine signals, maintains macrophage function, and shapes granuloma fate.

### Circulating Monocytes: Hyperactive Perpetuators of Granulomatous Inflammation

Monocytes are circulating innate immune cells that not only serve as precursors to monocyte-derived macrophages and monocyte-derived dendritic cells (moDC) but also play key roles in immune surveillance, inflammation, and tissue remodeling. Functionally, distinct subsets are defined by differential surface expression of CD14 and CD16, which correspond to classical (CD14 ^++^ CD16 ^−^ ), intermediate (CD14 ^++^ CD16 ^+^ ), and non-classical (CD14 ^+^ CD16 ^++^ ) monocyte populations. Classical monocytes (CD14 ^++^ CD16 ^−^ ) are dominant in the circulation and possess strong phagocytic potential, while intermediate (CD14 ^++^ CD16 ^+^ ) are expanded in inflammatory states and produce high levels of proinflammatory cytokines while also exhibiting antigen-presenting capacity, and non-classical (CD14 ^+^ CD16 ^++^ ) monocytes are involved in vascular surveillance and tissue repair. 65 66

Monocytes are increasingly recognized as key contributors to sarcoidosis pathogenesis, with alterations in their abundance, phenotype, and function observed across tissue compartments. Several groups have demonstrated a reduction in circulating classical monocytes, particularly among patients with untreated or progressive disease, and an expansion of intermediate monocytes in those with chronic, non-resolving phenotypes. 66 67 68 69 70 In contrast, monocytes and monocyte-derived cells are significantly increased in the alveolar space in frequency compared with healthy controls, 67 69 70 consistent with active recruitment into sites of active disease. Recruited monocytes are a major source of alveolar macrophages in sarcoidosis. 71 They exhibit increased expression of P2X7 receptors which enhances their ability to form MGCs, a hallmark of granulomatous inflammation. 72 These phenotypic and functional shifts position monocytes as pivotal drivers of the persistent antigen presentation and dysregulated inflammation that sustain granuloma formation in sarcoidosis.

Functionally, monocytes in sarcoidosis display an activated, proinflammatory phenotype. Circulating monocytes exhibit increased expression of PRRs such as TLR2 and TLR4 37 primed for activation, as well as markers of migration and activation, including CD16, CD69, VLA-1, and various chemokine receptors. 73 74 Intermediate monocytes, distinguished by 6-sulfo LacNAc expression for improved functional phenotyping, reveals increased *MHCII* expression consistent with heightened antigen-presenting capacity in sarcoidosis. 66 Furthermore, single-cell transcriptomic analyses further reveal enrichment in genes involved in trafficking, immune regulation, and inflammatory signaling, including mTOR, HMGB1, and ephrin receptor pathways, 74 indicating that circulating monocytes are not only pre-activated but also transcriptionally equipped to migrate into tissues and perpetuate local inflammation.

Monocyte recruitment to inflamed tissue is orchestrated by chemokines, CCL2, CCL7, and CCL20, which are upregulated in response to proinflammatory stimuli, as well as by cytokines such as IFN-γ and TNF-α, both of which are elevated in the granulomatous microenvironment. 75 76 Monocytes in sarcoidosis demonstrate enhanced migratory potential, shaped by dynamic regulation of chemokine receptors across compartments. CCR2, the receptor for CCL2, is highly expressed on classical monocytes and promotes recruitment into inflamed tissue, SNPs of which are associated with increased sarcoidosis risk. 77 While circulating classical monocytes express minimal CCR7, this receptor is markedly upregulated in monocytes from BALF and endobronchial biopsies, suggesting localized acquisition of lymph node–homing potential and tissue-specific immune programming. 69 Other trafficking molecules, including CD11b and integrins, are similarly upregulated. 73 Once recruited into the lung or lymphatic tissue, monocytes differentiate into monocyte-derived macrophages or DCs, where they actively contribute to granuloma formation, cytokine production, and T cell activation. 78 This dynamic recruitment and differentiation process not only supports granuloma architecture but also sustains local inflammation, particularly when antigen clearance is impaired. The central role of monocytes is further evidenced by the recurrence of sarcoidosis in transplanted lungs, underscoring their systemic contribution to disease pathogenesis. 79 80

Once in the lung, monocytes are potent amplifiers of inflammation. In BALF, they produce high levels of TNF-α in the absence of exogenous stimulation. 70 Notably, the frequency of TNF-producing monocytes/monocyte-derived cells in BALF at diagnosis is highest among patients who later develop progressive disease, implicating these cells in chronic immune activation and disease worsening. 70 Their proinflammatory function is further amplified by BALF exosomes, which are increased in sarcoidosis and induce monocyte production of IL-1β, IL-6, TNF-α, and CCL2 in a dose-dependent manner. 81 Compounding this activation is impaired negative regulation, as demonstrated by reduced expression of the inhibitory receptor CD200R, which is associated with enhanced TNF and IL-6 production, both at baseline and following stimulation. 82 These findings characterize a monocyte compartment that is both hyperresponsive and insufficiently restrained, positioning it as a key driver of persistent inflammation and fibrotic progression in sarcoidosis.

In contrast, monocytes isolated from lung-draining lymph nodes are less abundant and display a more immature, less activated phenotype, and reduced levels of maturation markers and migratory proteins compared with BALF and endobronchial biopsy samples. 69 83 These features are consistent with a more quiescent state, potentially shaped by a suppressive or regulatory lymph node microenvironment, and underscore the lung as the predominant site of monocyte activation and inflammatory programming in sarcoidosis.

Beyond inflammatory activation, monocytes in sarcoidosis exhibit altered phagocytic capacity. Increased expression of Fcγ receptors and reduced expression of complement receptors CR1 and CR4 suggest disordered handling of opsonized antigens and impaired clearance of inflammatory stimuli. 84 85 Transcriptional profiling of BAL monocytes demonstrates upregulation of genes associated with lysosomal processing and phagocytosis, alongside downregulation of proteasomal and ribosomal pathways, suggesting a shift toward sustained antigen processing. 86 87 More recently, altered regulation of key transcriptional repressors, including *TLE3* and *CBX8* , has been implicated in monocyte dysfunction, with downstream effects on CD4 ^+^ T cell depletion. 88 Together, these findings highlight a monocyte compartment that is not only overactivated but also deficient in regulatory and resolution mechanisms, perpetuating chronic inflammation and fibrosis in sarcoidosis.

### Dendritic Cells: The Bridge Between Innate and Adaptive Immunity

DCs are rare, comprising less than 1% of hematopoietic cells in blood and lymphoid tissues, 89 and highly specialized antigen-presenting cells that play a pivotal role in initiating and sustaining adaptive immune responses. Despite representing a small fraction of immune cells, DCs are among the most efficient antigen-presenting cells, 90 characterized by high expression of MHC class II molecules 91 and a potent ability to initiate and augment T cell responses. 92 93 In sarcoidosis, DCs contribute significantly to granuloma formation and maintenance, with evidence that both conventional and plasmacytoid DCs exhibit compartment-specific phenotypes and functions that shape granulomatous inflammation. A focused understanding of their spatial distribution, activation state, and crosstalk with T cells and macrophages is critical to uncovering their precise contributions to sarcoidosis pathogenesis.

At steady state, DCs, derived from common DC precursors, exist in multiple phenotypic subsets defined by distinct surface markers and functional capacities. Conventional DCs (cDCs), formally known as myeloid DC, express CD11c, and are further classified into cDC1 and cDC2 subsets. cDC1 cells are marked by *CLEC9A* and *XCR1* expression and promote Th1 responses, while cDC2 cells express SIRPα and CD1c, contributing to Th2 and Th17 polarization. 94 These cells express a broad array of TLR and become activated upon recognizing PAMPs or DAMPs, upon which they migrate to secondary lymphoid organs 95 where they present processed antigens to naïve CD4 ^+^ T cells via MHC class II, driving the differentiation of naïve T cells into effector subsets. 96 97 98 99 100 Plasmacytoid DCs (pDC), formally known as lymphoid DC, are found in blood and lymphoid organs and also exist at steady state. The cells are potent producers of type I interferons in response to viral nucleic acids and are increasingly implicated in autoimmunity due to inappropriate activation by self-antigens. Finally, upon initiation of a proinflammatory state, stimuli such as TNF-α promotes differentiation of moDC, also known as inflammatory DC, which express high levels of CD1a, CD11c, and HLA II surface molecules and contribute to Th1- and Th17-skewed responses, 101 102 which is highly relevant to sarcoidosis pathophysiology. Through their capacity to integrate innate sensing, antigen presentation, and cytokine-mediated T cell programming, DCs serve as key orchestrators of adaptive immunity. Their ability to direct T helper differentiation and perpetuate inflammatory cascades positions them as central players in granulomatous inflammation.

Experimental models demonstrate DCs are critical for the initiation and persistence of granulomatous inflammation. In a *Propionibacterium acnes* mouse model, administration of exogenous cDCs enhanced granuloma formation, which was reduced by CXCR3 and CCR5 blockade, 103 underscoring the importance of chemokine-driven DC recruitment. In a carbon nanotube model, ongoing DC-mediated antigen presentation and T cell activation were necessary to sustain granulomatous inflammation. 104 Collectively, these findings indicate that DCs drive granuloma formation and maintenance through antigen presentation, co-stimulation, and continuous recruitment of immune cells.

In sarcoidosis, DCs are found in increased numbers and exhibit a more mature, activated phenotype within lymph nodes and affected tissues. 105 106 Among these, cDCs are the predominant subset involved in granuloma formation. 105 106 Within affected tissues, cDCs are functionally upregulated, characterized by high immunocompetence and increased expression of costimulatory molecules, a proinflammatory profile with increased expression of inflammatory cytokines including IL-1, IL-6, and TNF-α. 107 108 109 Additionally, they reside in close proximity to T cells 109 and are capable of driving robust T cell proliferation and polarization toward Th1 and Th17.1 phenotypes, both of which contribute to granulomatous inflammation and disease progression. 105 110

Activated DCs are central to the propagation of Th1 responses through modulation of IFN-γ. They secrete IL-12, which increases T-bet ( *Tbx21* ) transcription factor expression through IFN-γ–mediated STAT1 activation. This in turn further enhances IFN-γ transcription, increases IL-12 receptor ( *IL-12RB2* ) expression, and suppresses *GATA3* , which is a key regulator of Th2 differentiation. 111 Additionally, IL-12 upregulates IL-18, which amplifies both IL-12R and IFN-γ signaling. Elevated levels of IL-12 and IL-18 have been found in BALF from patients with sarcoidosis, 112 113 underscoring the role of DC-driven Th1 skewing in disease pathogenesis.

Plasmacytoid dendritic cells (pDCs) are overall less abundant and serve an immune regulatory function mediated through IFN-γ signaling. 69 105 110 114 Notably, while pDCs are consistently detected in cutaneous sarcoidosis lesions, they exhibit reduced type I interferon production, suggesting impaired regulatory capacity. 114

In contrast to their tissue counterparts, both cDCs and pDCs are decreased in peripheral blood of patients with sarcoidosis compared with healthy controls, 69 109 115 suggesting active recruitment and dynamic trafficking to sites of inflammation. Moreover, peripheral cDCs demonstrate reduced functional capacity, with a diminished ability to stimulate allogeneic T cell proliferation despite upregulation of costimulatory markers, consistent with a state of functional anergy. 116 In contrast, moDCs exhibit proinflammatory function by stimulating increased TNF-α release when cultured with naïve CD4 ^+^ T cells. 105 These findings highlight the compartment-specific behavior of DC subsets in sarcoidosis and suggest that distinct tissue microenvironments play a critical role in shaping their phenotype and function.

### Dendritic Cell and Macrophage Feedback

DCs and macrophages engage in tightly regulated feedback mechanisms that help balance immune activation and suppression within the granuloma. Under normal conditions, macrophages suppress DC release of TNF-α, IL-12, and MMPs through PPAR-γ–mediated IL-10 production. In sarcoidosis, IL-10 release is inhibited by IFN-γ, thus allowing DCs to release proinflammatory cytokines and MMPs and contributing to tissue damage and disease progression. 117 Additionally, DC activate T cells, which stimulate macrophage differentiation into epithelioid and MGCs, further contributing to the granuloma core. 118 These reciprocal interactions between DCs and macrophages are central to granuloma organization and persistence, and their disruption may represent a key immunopathologic mechanism in sarcoidosis progression.

## Adaptive Immune Dysregulation

### CD4 ^+^ T Cell Activation

Sarcoidosis has long been considered a CD4 ^+^ T cell/Th1–mediated disease as CD4 ^+^ cells are central to the immunopathogenesis. They accumulate in affected organs, particularly the lungs, where they dominate the BAL compartment 119 and are critical to granuloma formation and maintenance. 120 Their selective enrichment, oligoclonality, and activated phenotype suggest a persistent, antigen-driven response, implicating them as key orchestrators of local inflammation and granuloma maintenance.

Naïve CD4 ^+^ T cells are activated in secondary lymphoid organs by antigen-presenting cells, primarily DCs and macrophages. This activation requires two signals, antigen recognition via the T cell receptor (TCR) binding to peptide–MHC class II complexes, and a costimulatory signal via CD80/CD86 binding to CD28 on the antigen-presenting cell. Together, these signals initiate clonal expansion, effector differentiation, and cytokine production, notably interleukin-2 (IL-2), which supports T cell proliferation and survival. 121 122 In addition, signals are significantly influenced by metabolic programming within the antigen-presenting cells, which further influence T cell fate decisions, including early memory programming and lineage commitment.

Effector CD4 ^+^ T cells in sarcoidosis patients display a limited and skewed TCR repertoire, indicating that a small number of T cell clones have expanded in response to specific antigens, often linked to specific MHC II polymorphisms, and correlating with disease activity. 123 124 125 126 Notably, increased proportions of CD4 ^+^ Vα2.3 ^+^ T cells in BALF have been shown to be highly specific for sarcoidosis, supporting sustained local T cell activation. 127 These T cells also express elevated activation markers and proliferate in response to sarcoidosis-associated antigens, supporting a role for specific exogenous or persistent antigens in driving the disease. 128

Beyond TCR specificity, sarcoidosis is marked by defects in key costimulatory and inhibitory signaling pathways. CD28-mediated co-stimulation, essential for cell cycle progression through PI3K/AKT signaling, is counteracted by the immune checkpoint receptor PD-1. 129 In progressive sarcoidosis, CD4 ^+^ T cells exhibit high PD-1 expression, which suppresses TCR-driven activation of the PI3K/AKT/mTOR pathway, impairing proliferation and cell cycle progression 130 and subsequently contributing to T cell exhaustion. Increased PD-1 expression on memory T cells predicted treatment response, 131 and PD-1 blockade restores signaling and proliferation in vitro. 132 These findings suggest that PD-1–mediated suppression of costimulatory signaling is a central feature of T cell dysfunction in sarcoidosis and may represent a promising target for therapeutic modulation in progressive disease.

### CD4 ^+^ T Cell Recruitment and Polarization

CD4 ^+^ T cells are actively recruited to local tissue through chemokine gradients, adhesion molecules, and chemoattractants secreted by macrophages. 61 133 Once localized, these T cells contribute to the inflammatory environment by secreting cytokines and chemokines, including GM-CSF, IFN-γ, and IL-23R, that reinforce macrophage activation and promote granuloma maintenance. 61 In early disease, T cell activity is particularly prominent, with tissue infiltrates enriched in IFN-γ, TNF-α, and IL-2–producing Th1 cells. 134 135 IL-2 supports continued CD4 ^+^ T cell expansion, while IFN-γ and TNF-α shape the inflammatory microenvironment required for granuloma persistence. This heightened effector response and substantial T cell accumulation within granulomas contrasts with the peripheral lymphopenia and anergic phenotype observed in circulation, reflecting a compartmentalized and dysregulated immune response. 88 116

T cells within the airways and affected tissues of sarcoidosis patients predominantly exhibit Th1, Th17, and Th17.1 phenotypes. 61 134 136 137 138 Ramstein et al identified an enrichment of Th17.1 cells in BALF of sarcoidosis compared with healthy controls. 136 More recently, spatial single transcriptomics confirmed Th17.1 cells, driven by IL-12 and IL-23, as the predominant CD4 ^+^ T cell subset within granulomas. These cells express elevated levels of *PDCD1* and *CTLA-4* , which encodes PD-1 and CTLA1, respectively, with concurrent downregulation of autophagy and cell cycle–related genes, which is consistent with chronic antigenic stimulation and an exhausted phenotype. At the protein level, these cells produce high levels of IFN-γ and GM-CSF, which serve as potent mediators of macrophage activation and myeloid cell recruitment. 61 Together, these features highlight Th17.1 cells as key effectors in sustaining the inflammatory milieu of chronic sarcoidosis. Together, these findings suggest that Th17.1 cells are central orchestrators of granulomatous inflammation in sarcoidosis, with dual roles in promoting both proinflammatory and regulatory signaling. Their functional plasticity, exhaustion markers, and antigen-specific activation highlight their potential as both biomarkers of disease chronicity and targets for immunomodulatory therapy.

### Peripheral CD4 T Cell Dysfunction

Circulating CD4 ^+^ T cells are reduced in sarcoidosis, likely reflecting a combination of recruitment to sites of inflammation, increased apoptosis, and disrupted homeostatic regulation. 74 88 139 140 Functionally, both naïve and effector T cell subsets exhibit alterations suggestive of systemic immune dysregulation that contributes to sarcoidosis.

Naive CD4 ^+^ T cells are reduced and primed toward an activation state in sarcoidosis. 74 139 In vitro stimulation of these cells reveals an altered cytokine profile characterized by increased IL-2 and TNF-α but decreased IFN-γ, along with reduced CD69 upregulation following TCR engagement, indicating a blunted or atypical activation response. 139 A subset of naïve T cells displayed increased CD25 expression, the α chain of IL-2 receptor, indicating heightened IL-2 responsiveness and early T cell activation that was also associated with chronic active disease at follow-up. 139 Additionally, scRNA-seq revealed upregulated expression of activation-associated pathways, including JAK/STAT, PI3K/AKT, and ERK/MAPK signaling, as well as dysregulation of apoptotic pathways and TGF-β/HIPPO signaling, which govern Th17 and Treg differentiation. 74

Effector T cells are also functionally impaired. Peripheral early effector T cells demonstrate downregulation of TCR and ICOS-ICOSL signaling, as well as suppression of PI3K/AKT and mTOR pathways, consistent with an anergic or hyporesponsive phenotype. 74 Similarly, Oswald-Richter et al reported diminished TCR-mediated activation in peripheral CD4 ^+^ T cells, including reduced signaling through IL-2, Lck, NF-κB, and Src kinases, further supporting the presence of peripheral T cell anergy in sarcoidosis. 137 Together, these findings suggest that in sarcoidosis, circulating CD4 ^+^ T cells are numerically reduced and functionally dysregulated, with naïve cells primed toward activation and effector differentiation, while peripheral effector T cells display features of anergy. This dual dysfunction possibly contributes to chronic immune activation and impaired granulomatous resolution in sarcoidosis.

## Decreased Immunoregulatory Mechanisms

### Regulatory T Cells

Regulatory T cells (Tregs), defined by the expression of FOXP3 and CD25, constitute approximately 5 to 10% of the circulating CD4 ^+^ T cell population and are essential for maintaining immune tolerance and suppressing excessive effector T cell response. They function by suppressing autoreactive T cells and limiting excessive inflammatory responses, thereby preserving immune homeostasis. 141 Disruption of Treg function can lead to immune dysregulation, as evidenced by autoimmune disease in the absence of adequate Treg activity or mutations. 142 143

Tregs are implicated in the immune dysregulation underlying sarcoidosis. Most studies report a numerical expansion of Tregs, with increased frequencies observed in peripheral blood, BALF, and within granulomatous tissue in patients with active or chronic disease, 144 145 146 147 though studies are not consistent. These Tregs typically express canonical suppressive markers including FoxP3, CD25, and CTLA-4. 144 Treg number correlates with markers of immune activation, such as thoracic lymphadenopathy and increased systemic symptom burden, but shows no consistent association with fibrosis or radiographic stage. 147 148 Huang et al, however, reported decreased Treg numbers in both peripheral blood and BAL, accompanied by a reciprocal increase in Th17 cells and an elevated Th17:Treg ratio. 149 These findings normalized with corticosteroid therapy, suggesting that Treg and Th17 frequencies are dynamic and responsive to immunosuppression. The reduction in Tregs observed in this cohort may also reflect underlying genetic influences, as Wikén et al previously demonstrated decreased Treg numbers in the BAL of sarcoidosis patients carrying the HLA-DRB1*0301 allele. 150

Despite their numeric expansion, Tregs in sarcoidosis fail to adequately suppress inflammatory cytokine production, permitting persistent immune activation and granuloma formation. In affected tissues, Tregs exhibit reduced suppressive capacity, shortened telomeres consistent with proliferative exhaustion, and aberrant secretion of proinflammatory cytokines such as IL-4, which may contribute to fibroblast activation and granuloma persistence. 151 In mediastinal lymph nodes, decreased CTLA-4 expression on both Tregs and Th17 cells may impair local regulatory control and promote unchecked Th17-driven inflammation. 152 In the periphery, Tregs also exhibit increased CD95 expression and are more prone to apoptosis, further limiting their persistence and effectiveness. 144 Patterson et al assessed peripheral blood Tregs using flow cytometry and suppression assays, finding preserved in vitro function but reduced in vivo activity among patients with mediastinal lymphadenopathy or high symptom burden. 148 Transcriptomic analyses of PBMC further reveal downregulation of *BACH2* and *NR1D1* , transcription factors critical for Treg differentiation and concurrent Th17 suppression, in cases with advanced organ involvement and severe pulmonary disease. 153 Together, these findings highlight a state of functional Treg insufficiency that may permit chronic inflammation, tissue remodeling, and progression to fibrosis.

Importantly, Treg dysfunction appears to associate more strongly with chronic and fibrotic disease phenotypes than with acute inflammation. Miedema et al used multi-color flow cytometry and longitudinal follow-up to identify a distinctive Treg phenotype associated with chronic sarcoidosis, characterized by increased expression of CD25, CTLA-4, CD69, PD-1, and CD95. 139 These findings suggest that dysfunctional Tregs not only persist in chronic disease but may acquire an activated yet ineffective phenotype. Taflin et al further demonstrated that tissue Treg number did not correlate with granulomatous inflammation but was positively associated with interstitial fibrosis in renal sarcoidosis, supporting a role for regulatory failure in fibrotic remodeling. 147 Moreover, in vitro studies showed that Treg depletion enhanced granuloma formation in healthy donor cultures but had no effect in sarcoidosis-derived cells, underscoring the presence of disease-associated Treg dysfunction. Restoration of Treg and Th1 cell function has been observed in patients with spontaneous clinical resolution, highlighting the importance of intact regulatory mechanisms in disease control. 137 Taken together, these data support a model in which Tregs in sarcoidosis are not simply reduced or expanded but are functionally impaired in ways that allow for ongoing immune activation, granuloma persistence, and possibly fibrotic progression.

### Role of iNKT Cells

Invariant natural killer T (iNKT) cells are a specialized subset of T lymphocytes that co-express TCR and surface markers typically associated with natural killer (NK) cells. Unlike conventional T cells, iNKT cells recognize lipid antigens presented by the non-polymorphic, MHC class I–like molecule CD1d, allowing them to respond rapidly to stress signals and pathogen-associated lipids. Upon activation, iNKT cells secrete a broad array of cytokines, including IFN-γ, IL-4, and IL-10, and play important roles in immunoregulation, immune surveillance, and the modulation of inflammatory and autoimmune responses. 154 155 156 Two main subsets exist: CD1d-dependent invariant NKT cells (iNKT cells), which possess immunoregulatory properties, and CD1d-independent NKT-like cells with more variable phenotypes.

Multiple studies have shown that iNKT cells are reduced in the peripheral blood and BALF of sarcoidosis patients, particularly in those with chronic or progressive disease. Markedly diminished levels have been observed in circulation and are often absent in mediastinal lymph nodes, granulomas, and cutaneous lesions. 157 158 In the lung, reduced iNKT cell frequencies in BALF negatively correlate with CD4 ^+^ T cell abundance, suggesting a potential role in regulating local T cell expansion. 159 In contrast, patients with acute, self-limited disease such as Löfgren syndrome often exhibit preserved or increased iNKT cells in BALF, a pattern associated with better prognosis. 159 160

iNKT cells also exhibit functional impairments marked by diminished cytokine secretion and reduced regulatory capacity. This dysfunction is especially evident in chronic disease, where circulating iNKT cells produce less IFN-γ, indicating a loss of Th1-driven immune modulation. 157 160 Loss of iNKT cells in sarcoidosis has been associated with impaired monocyte-derived IL-10 production and reduced suppression of T cell proliferation, deficits that are reversible with iNKT cell reconstitution in vitro. 161 Functionally, the absence of IFN-γ and dual IFN-γ/TNF-α iNKT subsets correlates with markers of disease severity, including reduced forced vital capacity, elevated C-reactive protein, and radiographic evidence of fibrosis. 162

These findings suggest that iNKT cell loss and dysfunction contribute to the exaggerated T cell activation, deficient immune regulation, and persistence of inflammation that characterize sarcoidosis. Preservation of iNKT cell number and function in resolving disease further supports their role as modulators of disease course and severity.

## Additional Implicated Cell Types

Although macrophages are at the granulomatous core, surrounded and supported by recruited CD4 ^+^ T cells and monocyte precursors, multiple other immune and structural cell types are present within granulomas and affected tissues. These include CD8 ^+^ T cells, B cells, NK cells, as well as structural cells such as fibroblasts and endothelial cells. Studies reveal numerous phenotypic and functional differences in these populations compared with healthy controls, but it remains unclear whether these changes reflect primary dysregulation or a secondary response to upstream immune activation.

CD8 ^+^ T cells, or cytotoxic T lymphocytes, are essential for antiviral and antitumor immunity through direct cytotoxic killing, and their abnormal expansion and hyperactive effector function have been implicated in various autoimmune diseases. 163 In sarcoidosis, CD8 ^+^ T cells are expanded in circulation, with higher levels correlating with worse clinical outcomes. 164 165 166 BAL shows reduced CD8 ^+^ abundance, and a high CD4 ^+^ :CD8 ^+^ ratio is sometimes used to help distinguish sarcoidosis from alternate diagnoses, albeit with limited sensitivity. 167 In contrast, cerebrospinal fluid from patients with neurosarcoidosis demonstrates clonal expansion of CD8 ^+^ T cells, underscoring the tissue- and phenotype-specific nature of CD8 ^+^ T cell responses. 168 Phenotypically, CD8 ^+^ T cells exhibit a Th1-skewed profile with elevated IFN-γ and TNF-α 164 166 and overexpress activation, adhesion, and senescence markers, consistent with persistent antigen-driven activation, similar to CD4 ^+^ T cells, highlighting the role of the microenvironment in shaping effector function. 169 Genetic associations with CD8-related variants in Löfgren's syndrome suggest a role for CD8 ^+^ biology in clinical phenotype variation. 170 Mechanistically, progressive disease involves aberrant SHP2 signaling in CD8 ^+^ T cells, which disrupts SKP2-mediated TBET ubiquitination, driving sustained TBET activity, excessive IFN-γ production, and macrophage-mediated fibrosis. CD8 ^+^ T cells are therefore important in granuloma persistence, disease severity, and progression.

B cells, characterized by CD20 surface expression, are adaptive immune lymphocytes that bridge humoral and cellular immunity. They function as professional antigen-presenting cells to activate T cells and, upon activation, can differentiate into memory B cells or antibody-secreting plasmablasts and plasma cells. Additionally, B cells influence immune regulation through cytokine secretion and modulation of T cell responses, including both effector and regulatory pathways. In sarcoidosis, B cells and IgA-producing plasma cells localize to the outer layer and between granulomas. 171 172 In circulation, naïve, activated, and regulatory B cells are expanded, whereas memory B cells are reduced. 173 174 T follicular helper cells, which drive B cell activation, are also increased and express markers of enhanced B cell crosstalk. 173 175 Chronic stimulation is evident from elevated plasma B cell activating factor (BAFF) and hypergammaglobulinemia across multiple phenotypes. 176 177 Age-associated B cells, a distinct subset of B cells that arise from chronic antigenic stimulation and display proinflammatory function, are additionally expanded in sarcoidosis and are responsive to treatment. 178 B cells also exhibit features of exhaustion and produce immunoglobulins with high frequencies of somatic hypermutation and increased downstream IgG subclass usage, consistent with prolonged or repetitive antigen exposure. Although B cell depletion with anti-CD20 monoclonal antibodies has shown clinical benefit in refractory disease, 179 180 the extent to which B cell alterations directly drive granuloma persistence remains unclear.

NK cells are innate lymphocytes whose functions correlate with CD56 expression: CD56 ^bright^ cells primarily regulate immune responses through cytokine secretion, whereas CD56 ^dim^ cells provide rapid cytotoxic defense against infected and malignant cells. In sarcoidosis, lung NK cells are enriched for the CD56 ^bright^ subset, while CD56 ^dim^ cells predominate in blood. 181 Higher NK cell proportions in BAL are associated with worse clinical outcomes and greater need for treatment, 182 183 yet their overall abundance is lower than in other fibrotic interstitial lung diseases. 184 Conversely, reduced NK cell percentages in peripheral blood have been linked to cardiac involvement, 185 suggesting NK cell abundance varies by disease phenotype and may reflect organ-specific recruitment or regulation. Pulmonary NK cells produce high levels of IFN-γ and TNF-α, contributing to the Th1-polarized inflammatory environment of granulomas. 181 These findings suggest NK cells in sarcoidosis adopt a cytokine-producing, proinflammatory phenotype that may sustain local inflammation rather than serving solely in cytotoxic clearance.

Finally, structural cells, including endothelial cells and fibroblasts located at the granuloma border, are increasingly recognized as active participants in sustaining granulomatous inflammation. In sarcoidosis, endothelial cells promote ECM remodeling, focal adhesion, and immune cell migration, while granuloma-associated fibroblasts display an inflammatory phenotype characterized by macrophage activation and recruitment, antigen presentation, and TGF-β–mediated signaling, as well as a tissue-remodeling phenotyping that supports ECM and angiogenesis. 61 Together, these cells coordinate the structural and immunologic support needed to maintain chronic granulomatous inflammation.

Other immune and stromal cell types may also contribute to granuloma initiation and maintenance but are outside the scope of this review.

## Conclusion

Sarcoidosis is a multisystem, heterogeneous disease arising from an aberrant immune response to an unidentified antigen in genetically susceptible individuals. It results in a well-organized, non-caseating granuloma consisting of tightly clustered epithelioid histiocytes and MGCs, encircled predominantly by CD4 ^+^ T lymphocytes and fibroblasts, with additional contributions from CD8 ^+^ T cells and NK cells. Granuloma development and persistence reflect the coordinated activity of these immune cell populations. Macrophages and DCs initiate and sustain granulomatous inflammation through antigen presentation, cytokine production, and recruitment of monocytes, which differentiate into epithelioid cells, MGCs, and inflammatory DCs. Within granulomas, CD4 ^+^ T cells, particularly Th1, Th17, and Th17.1 subsets, are oligoclonally expanded and exhibit a phenotype consistent with chronic antigenic stimulation and partial exhaustion, driving ongoing inflammation. Impaired Treg cell function, loss and dysfunction of iNKT cells, and enrichment of other immune effectors including cytotoxic CD8 ^+^ T cells, activated B cells, NK cells, and tissue-resident structural cells further amplify and perpetuate the inflammatory milieu.

Despite advances in defining the cellular and molecular landscape of sarcoidosis, key questions remain unanswered. The initiating antigens, the mechanisms linking systemic immune activation to diverse clinical phenotypes, and the determinants of resolution versus progression to fibrosis are still incompletely understood. Future studies integrating high-resolution spatial, single-cell, and functional approaches will be essential to disentangle primary drivers from secondary changes, clarify the interplay between immune and structural cells, and identify actionable pathways that could alter the natural history of this enigmatic disease.
